# Feasibility study of the Nox-T3 device to detect swallowing and respiration pattern in neurologically impaired patients in the acute phase

**DOI:** 10.1038/s41598-023-32628-y

**Published:** 2023-05-05

**Authors:** Fanny Theytaz, Aline Vuistiner, Valérie Schweizer, Adélie Crépin, Kishore Sandu, Aziz Chaouch, Lise Piquilloud, Gianpaolo Lecciso, Kay Coombes, Karin Diserens

**Affiliations:** 1 University of Lausanne, 1015 Lausanne, Switzerland; 2 Hôpital Fribourgeois, 1752 Villars-sur-Glâne, Switzerland; 3 Phoniatrics and Speech Therapy Unit, Lausanne University Hospital (CHUV), Rue du Bugnon, 46, 1011 Lausanne, Switzerland; 4 Centre Hospitalier du Valais Romand, 1951 Sion, Switzerland; 5 Airway Unit, Department of Otorhino-Laryngology and Head and Neck Surgery, Lausanne University Hospital (CHUV), Rue du Bugnon, 46, 1011 Lausanne, Switzerland; 6 Division of Biostatistics, Center for Primary Care and Public Health (Unisanté), University of Lausanne, Lausanne, Switzerland; 7 Adult Intensive Care Unit, Lausanne University Hospital (CHUV), Rue du Bugnon, 46, 1011 Lausanne, Switzerland; 8 Centre d’investigation et de Recherche sur le Sommeil, Rue du Bugnon 46, 1011 Lausanne, Switzerland; 9 ARCOS, Malvern Centre, Hatherton Lodge, Avenue Road, Malvern Worcestershire, WR14 3AG UK; 10 Acute Neurorehabilitation Unit, Neurology Service, Department of Clinical Neurosciences, Lausanne University Hospital (CHUV), Rue du Bugnon, 46, 1011 Lausanne, Switzerland

**Keywords:** Neurology, Oral diseases, Neurological disorders, Disorders of consciousness, Rehabilitation

## Abstract

Dysphagia is a frequent complication in neurologically impaired patients, which can lead to aspiration pneumonia and thus prolonged hospitalization or even death. It is essential therefore, to detect and assess dysphagia early for best patient care. Fiberoptic endoscopic and Videofluoroscopy evaluation of swallowing are the gold standard exams in swallowing studies but neither are perfectly suitable for patients with disorders of consciousness (DOC). In this study, we aimed to find the sensitivity and specificity of the Nox-T3 sleep monitor for detection of swallowing. A combination of submental and peri-laryngeal surface electromyography, nasal cannulas and respiratory inductance plethysmography belts connected to Nox-T 3 allows recording swallowing events and their coordination with breathing, providing time-coordinated patterns of muscular and respiratory activity. We compared Nox-T3 swallowing capture to manual swallowing detection on fourteen DOC patients. The Nox-T3 method identified swallow events with a sensitivity of 95% and a specificity of 99%. In addition, Nox-T3 has qualitative contributions, such as visualization of the swallowing apnea in the respiratory cycle which provide additional information on the swallowing act that is useful to clinicians in the management and rehabilitation of the patient. These results suggest that Nox-T3 could be used for swallowing detection in DOC patients and support its continued clinical use for swallowing disorder investigation.

## Introduction

Swallowing is a complex process that aims to provide nutrition while protecting the airways. The bilateral synchronized actions of muscles allows the delivery of a bolus from the oral cavity to the stomach while maintaining adequate protection of the airways^1,2^. This process is divided into three phases: oral, pharyngeal and esophageal. The oral phase is a voluntary act and may be interrupted at any time. Then, once the bolus reaches the oropharynx it triggers the swallowing reflex carrying out the pharyngeal and esophageal phases^1,3^. These mechanisms are controlled by the brain stem central pattern generators (CPG) (6,7). The complex nervous organization of swallowing is summarized by the concept of Broussard et al.^4^, the CPG receives information from sensory fibers from the periphery and the cortical and subcortical descending inputs also end here^5^. The cortex is therefore also important for swallowing by voluntarily initiating or modulating it^2,6^.

Respiration plays an essential role in swallowing. Both have to be coordinated in order to limit the risk of aspiration^7–9^. It is possible to swallow at any moment, but most of the time spontaneous swallowing takes place during the expiratory cycle^10,11^. Moreover, there is a pause in the respiratory cycle during deglutition, called the swallowing apnea, that lasts from 1 to 3 s^10,12,13^. During apnea laryngeal closure occurs in order to protect the airway from aspiration. After this central apnea, the respiratory cycle resumes with an expiratory phase, regardless of when swallowing was initiated^8,14^.

This study focuses on dysphagia in patients with disorders of consciousness (DOC) resulting from stroke or traumatic brain injury (TBI). DOC patients can range from unresponsive wakefulness syndrome (UWS) to minimally conscious state (MCS) but also patients with cognitive motor dissociation (CMD)^15^. Dysphagia is one of the life-threatening complications in DOC patients, with a prevalence following stroke varying from 8.1% to 45.3% and from 27 to 30% following TBI, depending on the study^16,17^. Dysphagia can result from lesions at multiple levels. Coordination between respiration and swallowing may also be affected thus reducing airway protection. Dysphagia itself can lead to airway obstruction, aspiration-pneumonia, dehydration and malnutrition, which may lead to prolonged hospitalization or even death^18^. It is therefore crucial to evaluate dysphagia and coordinated respiratory patterns as early as possible in order to assure better outcome and prevent complications^19–21^. In addition, many studies have shown that swallowing initiation can be an early indication of conscious behavior and may provide evidence of conscious awareness in DOC patients^22–24^.

A variety of techniques are used to assess dysphagia and aspiration risk. Among others, Video fluoroscopy (VFS) and fiberoptic endoscopic evaluation of swallowing (FEES) are considered as the leading instrumental swallowing assessment methods^25,26^. Many clinical bedside evaluations are available^27^, such as the estimation part of the Facial Oral Tract Therapy (FOTT)^28,29^. However, there is no consensus in the literature of a standard reference for clinical evaluation^20^. Moreover, clinical assessments are only 66% accurate in detecting aspiration^30^. It is therefore necessary to complement this rating with an objective examination of swallowing^21^.

In order to provide an objective swallowing evaluation at bedside, different methods have been developed. Acoustic method^31,32^, electromyography and motion technics^33–35^ are used but there is currently no consensus on their use, especially in DOC patients. For this reason, the aim of this study is to propose a convenient method for detecting swallowing and respiration patients with swallowing disorders following neurological impairment. This method is intended to complement the bedside assessment by providing a trace of the swallowing act and adding information about the coordination between swallowing and breathing. The evaluation criteria were: The method should (1) be able to be performed at the patient's bedside; (2) be minimally invasive and quick to perform; (3) simultaneously record both respiration and swallowing. The main challenge is to find an adequate method that is sufficiently sensitive and specific. For this purpose, we decided to test the Nox-T3 portable sleep monitor, which has previously been used for sleep apnea assessment^36,37^. This device seems capable of meeting part of the above-mentioned criteria as it is a portable and non-invasive tool allowing the measurement of muscular and respiratory parameters. Our goal was to provide an objective measure of sensitivity and specificity of Nox-T3 for detection of swallowing and associated respiration pattern. This in order to validate de use of Nox-T3 for assessment of swallowing by DOC patients.

## Materials and methods

This is a non-randomized prospective study that enrolled 14 consecutive DOC patients admitted to the Acute Neuro-Rehabilitation Unit (NRA—Clinical Neurosciences Department, University Hospital of Lausanne, Switzerland), between November 2018 and March 2020. The local ethics committee, “Commission cantonale d'éthique de la recherche sur l'être humain—VD” (CER-VD) approved the experimental protocol (142/09) and all patients’ legally authorized representatives provided written informed consent. All methods were carried out in accordance with relevant guidelines and regulations. The criteria for inclusion in the study corresponded to those for NRA admission. Patients were screened by a neurologist specialized in neuro-rehabilitation and as follows:

- Early Rehabilitation Barthel Index (ERBI) < 30 at admission^38^
- With potential for rehabilitation, that was assessed weekly
- Can withstand the 300 min of treatment planned per day
- A diagnosis of coma awakening or neurological and/or surgical pathology, which requires a continuous care infrastructure, including the following criteria
  - Need for monitoring of vital signs and/or neurological signs ≥ 6 times/day
  - Need for respiratory support with non-invasive ventilation in a stable patient
  - Need for behavioral monitoring deemed insufficient in room
  - Other criteria: special equipment, continuous intravenous medication, etc.

For reasons related to the study, patients with the following characteristics were excluded:

- Known, pre-existing swallowing disorders
- Known, pre-existing neuromuscular diseases
- Medical contraindications involving fasting or the need to maintain a supine position
- Refusal to participate

The Nox-T3 portable sleep monitor (Nox-T3 System with WristOx2, Nox Medical, Reykjavik, Iceland, N°511010) used in this study in attempt to detect swallowing is currently validated for the detection of obstructive sleep apnea^36,37^. The company gave its approval for its use in this study. The Nox-T3 device gathers data through four to seven channels. For this study we recorded the following parameters:

- Nasal or tracheotomy airflow
- Respiratory effort through thoracic and abdominal movements
- Pulse oximetry
- Voice of the speech therapist by microphone

In addition, the two bipolar channels on the device were used for surface electromyography (sEMG).

### Nasal cannula

Direct nasal airflow was measured using a nasal cannula inserted at the entrance of the nostrils (Nox Cannula with Luer-lock, Nox Medical, Reykjavik, Iceland, N°552,020) and connected to the pressure lock of the Nox-T3 device by a filter tube connector (Nox Filter Tube Connector, ResMed Suisse SA, N°552,110). The airflow causes changes in pressure, inspiration causes negative pressure and expiration positive pressure^32,39^. These changes are detected via a pressure transducer that produces a time-related signal, recorded at a sampling frequency of 200 Hz and expressed in cmH_2_0. For patients with tracheotomy, if possible, we would use an airtight cap and take a nasal measurement. If this was not possible, an adaptor (Differential pressure transducer adapter, ResMed Suisse SA Nox, N° 1420,221) was used to detect the airflow through the tracheotomy tube. The technique to record changes in airflow remains the same as for nasal records.

### Respiratory inductance plethysmography (RIP) belt

**Figure 1:**
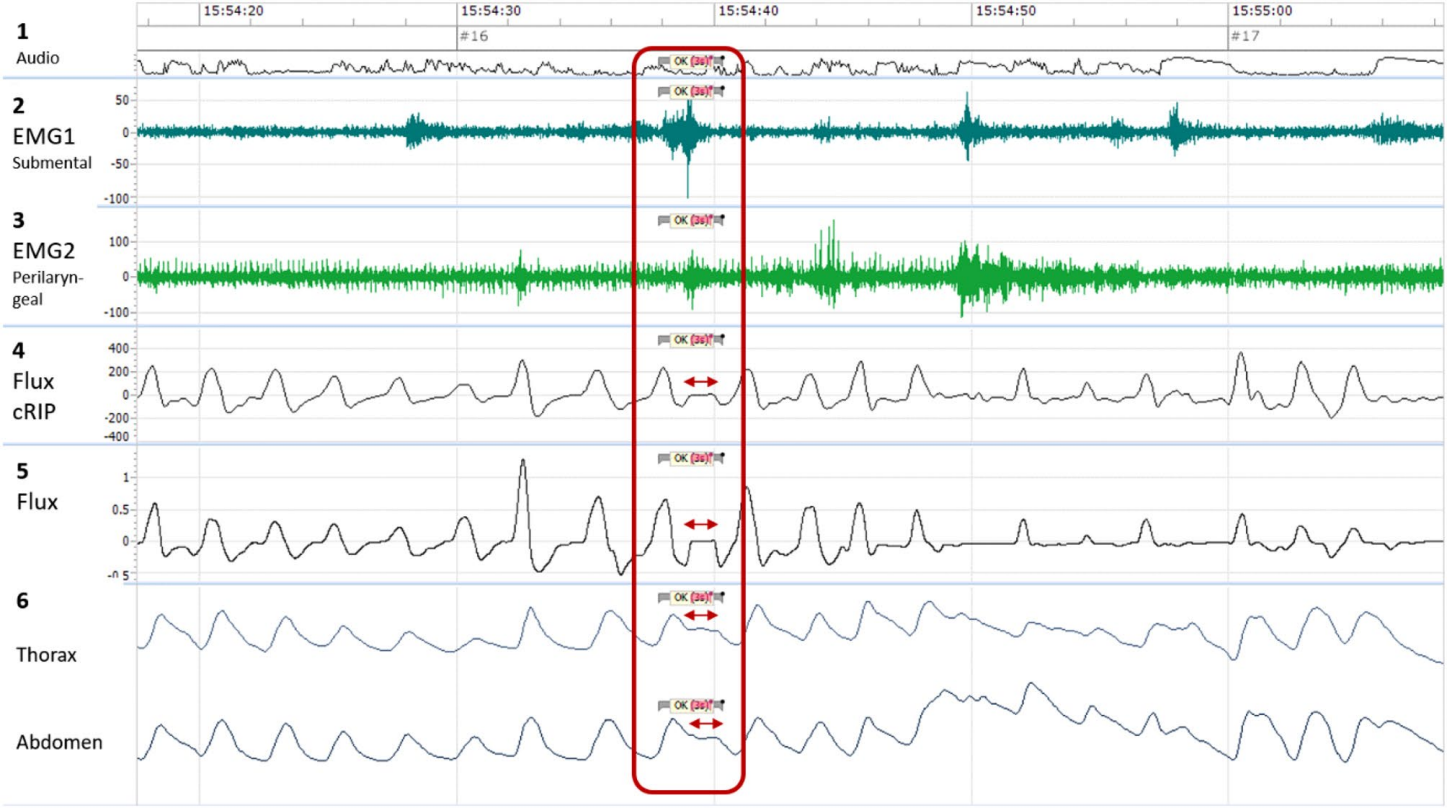
Nox- T3 signal. Graphical presentation of the signals obtained by Nox-T3 through sEMG submental and perilaryngeal, nasal airflow and RIP. The red boxed-area indicates a swallowing act. The arrows indicate the central swallowing apnea. Tract 1: Volume audio, corresponding to the voice of the therapist. Tract 2: EMG1 submental µV, corresponding to submental muscle activity. Tract 3: EMG2 perilaryngeal µV, corresponding to perilaryngeal muscle activity. Tract 4: Flux cRIP µV, corresponding to respiratory effort. Tract 5: Flux cmH2O, corresponding to nasal or tracheostomy airflow. Tract 6: Thorax and Abdomen µV/cm, corresponding to thoracic and abdominal movements.

Respiratory effort recording is essential in a swallowing study to supplement the nasal airflow respiratory measurement^39^. The thoracic and abdominal movements were monitored by RIP. This technique was developed by Cohen et al.^40^. Two belts (Nox RIP Belts, small/medium/large, ResMed Suisse SA, N° 551020/30/40) were positioned over the patient's clothes, one at the nipple level and the other at the umbilical level, both connected to Nox-T3. The movements were recorded at a sampling frequency of 20 Hz. As body movements may introduce artefacts, the signals were high pass filtered with a 2 Hz filter. The result is a clear real-time signal that represents the respiratory phases of inspiration and expiration respectively with inflection of the curve upwards and downwards (Fig. 1).

### Surface electromyography

We selected two superficial muscle groups to represent the oral and pharyngeal phases of swallowing. We utilized electrodes (Grass Reusable EEG “Tangle Free” Cup Electrodes, Natus Neurology SA, 100 cm gold, N°019–477500) connected to Nox-T3 to detect the activity of these muscle groups. The oral phase is represented by submental (SM) muscles, which include the geniohyoid innervated by C1, the mylohyoid and the anterior belly of digastric muscle innervated by CN V, all covered with platysma^41^. Electrodes for the oral phase were positioned bilaterally (see Fig. S2) and form the EMG1 signal. The pharyngeal phase is unilaterally represented by the perilaryngeal (PL) muscles, which include the sternohyoid, the sternothyroid, the thyrohyoid and the omohyoid^42,43^. Electrodes for the pharyngeal phase were unilaterally positioned (see Fig. S2) and form the EMG2 signal. Naturally, as swallowing act are continuous, we are conscious that these muscle groups do not only participate in one phase but their activities are overlapping.

The sEMG signals were recorded at a sampling frequency of 200 Hz. Impedance arising from the skin, cross-talk with other muscles and heartbeat create artefact noise on the signals^41^. To reduce skin impedance, we first cleaned the skin using cleaning paste (Conducting paste CHUV, CHUV Pharmacy, n° D000120) then the electrodes were filled with conductive paste (Ten20 Conductive EEG Paste, Nox Medical, Reykjavik, Iceland, N°555,020) and attached to the skin. Moreover, after the acquisition the two signals were filtered with a low pass filter of 10 Hz and a sector filter of 50 Hz, in order to display a clear signal and allow better interpretation. The data are expressed in microvolts and are the summed activity of the above muscle groups, represented in two plots by traces 2-EMG1 and 3-EMG2, allowing the interpretation of swallowing events as shown in Fig. 1. During the muscle contraction, a burst activity can be observed on the tracts.

### Signal synchronisation and software Noxturnal

To synchronize the different signal sources, we connected all of them to the Noxturnal software (Noxturnal software, ResMed SA) for data acquisition. The individual signals were filtered and scaled for manual analysis. As we mostly focused on the time sequence of the swallowing events, the amplitude of the signals (Volt) had no particular calibration. Each signal is thus represented as a plot, which are vertically superimposed, allowing an adequate temporal analysis as shown in Fig. 1.

### Protocol

We performed the procedure as early as possible after the patient’s admission to the NRA unit, from day 0 to day 3. Each patient had a single recording session as described below. First, we installed the Nox-T3 on the patient (Fig. S1). Then, over 10 min, the Nox-T3 recorded swallowing and respiration parameters while the speech therapist performed a manual palpation, according to the method describe by Woisard-Bassols^44^, to detect swallowing acts. When swallowing occurred, the speech therapist mentioned it aloud, recording it on the Nox-Microphone. During the next 10 min of Nox-T3-recording, a rehabilitation session according to the FOTT was done simultaneously as the manual palpation. The speech therapist also assessed the patient with the Functional Oral Intake Scale (FOIS)^45^. Finally, 10 min of Nox-T3-recording with simultaneous manual palpation, exactly the same as the first 10 min, was performed. Once the 30 min of recording were over, we transferred data from Nox-T3 to the Noxturnal software and all detectable swallowing acts and time frames were recorded and subjected to numerical analysis. (see detailed protocol in the supplementary data).

### Nox-T3 data analysis

To assess the inherent validity of the Nox-T3 evaluation, we compared it to a standard method, the manual palpation. The observation period for each patient was the sum of times 1 to 3, corresponding to 30 min, which were divided into 5 s intervals (i.e. 360 time-intervals of 5 s) for statistical analysis.

### Clinically observed (i.e. true) swallowing times

In the absence of a validated technique, manual palpation was considered in this study as the usual method of reliably detecting a patient’s swallowing act. The palpation was performed by a therapist with more than 10 years of experience. Some of the assessments were performed with a second therapist present for visual control.

For a given patient, we identified the times at which a true swallowing was observed (after manual palpation) by the voice recording on the microphone. When pairs of successive swallowing were separated by less than 5 s, only the first one was retained. The second was considered pathological movement related to the first swallowing, and the entire act was called “multiple swallowing”.

To avoid detection of incomplete swallowing, the therapist performing manual palpation did not mention a swallowing act until it is completely finished. The time at which swallowing is clinically detected (i.e. voice signal) refers then to the end of the swallowing act. There is therefore a delay between the onset detection of the swallowing act by Nox-T3 corresponding to the beginning of the act, and the clinical detection corresponding to the end.

### Nox-T3-detected swallowing times

Nox-T3 detection of swallowing act was derived from information contained in 3 signals: EMG1 corresponding to SM muscle activity, EMG2 corresponding to PL muscle activity and apnea. The presence of a signal was assessed visually, by one therapist, after training by technicians from Resmed, the supplier of the device. Eight different signal combinations, from A to H, are possible and are listed in Table 1. After clinical consideration we assumed that a positive Nox-T3 signal (i.e. corresponding to a completed swallowing act) would only occur if any or both of the 2 following conditions were satisfied:

- Presence of an EMG2 signal (irrespective of the presence/absence of other signals)
- Joint presence of EMG1 and apnea signals (irrespective of the presence/absence of EMG2; Absence due to a capture problem)

**Table 1:** 8 signal combinations from A to H. 0 = absence of signal; 1 = presence of signal.

	Swallowing detection					Non-swallowing		
	A	B	C	D	E	F	G	H
EMG1	1	0	1	1	0	0	1	0
EMG2	1	1	0	1	1	0	0	0
Apnea	1	1	1	0	0	1	0	0

### Definition of true and false positives or negatives

To calculate the sensitivity and specificity of the Nox-T3 method and assess its ability to reliably detect patient swallowing, the data were compared with those from manual palpation. It is first necessary to calculate the number of true positives (TPs), false positives (FPs), true negatives (TNs) and false negatives (FNs) for Nox-T3 signal over the 360 time-intervals for each patient. The four situations are summarized in Table 2 below.

**Table 2:** **:** Definition of TP, FP; TN; FN as correlation between Nox-T3 swallowing detection and clinical swallowing palpation.

True positive (TP)	False positive (FP)
✓ Nox-T3: A; B; C; D; E	✓ Nox-T3: A; B; C; D; E
✓ Clinical detection	❌ No clinical detection

True negative (TN)	False negative (FN)
❌ Nox-T3: F; G; H	❌ Nox-T3: F; G; H
❌ No clinical detection	✓ Clinical detection

### Statistical analysis

A statistician performed the statistical analyses using counts of TP, FP, TN and FN per patient to reconstruct individual (repeated) binary data for presence (1) or absence (0) of true swallowing, and presence (1) or absence (0) of a Nox-T3 signal over the 360 time-intervals. A mixed effects logistic regression model was used. This model is used to model binary variables and repeated measures, here the presence or the absence of a swallowing act during the 360 time intervals, in order to predict a variable in this case sensitivity and specificity. The model was fitted to the individual (repeated) data in order to estimate the overall sensitivity and specificity of the Nox-T3 swallowing detection.

## Results

Over sixteen months, we successively analyzed 14 patients clinically and with Nox-T3. The demographic details are shown in Table 3. Five patients were women (35.7%) and nine men (64.3%). The mean age was 58 years (range from 28 to 84). Five patients (35.7%) had a tracheostomy but only three (21.4%) a respiratory recording through the tracheostomy. The etiologies of the altered state of consciousness were of vascular origin for 71.4% and the rest of TBI. The vascular origin include cardiac arrest, stroke and subarachnoid hemorrhage. Each patient was assessed with several scores the day of recording. The mean value for ERBI was -180 with minimum at -225 and maximum at -20. The mean value for the Coma Recovery Scale-Revised (CRS-R)^46^ was 10.73 with minimum at 4 and maximum at 23. About the diagnosis of state of consciousness, 10 patients were MCS, 3 UWS and one did not have disorder of consciousness. Twelve patients had a value of 1 on the FOIS. The remaining two patients had a value of two.

**Table 3:** Epidemiological data.

ID	Age	Sex	Aetiology	CRS-R	Presence of tracheotomy	Tracheal recording	Pneumonia	Enteral feeding
1	33	F	I Stroke	6	0	0	0	G
2	46	M	HIBI	15	0	0	1	NG
3	60	M	I Stroke	12	0	0	1	NG
4	59	M	HIBI	13	1	0	1	NG
5	51	F	ICH	10	0	0	0	NG
6	50	F	SAH	15	1	1	1	NG
7	61	M	TBI	7	0	0	1	G
8	73	M	I Stroke	23	0	1	0	NG
9	78	M	HIBI	4	0	0	1	NG
10	78	F	ICH	16	0	0	1	G
11	61	M	I Stroke	13	1	0	1	NG
12	84	M	TBI	5	0	0	1	NG
13	54	M	TBI	6	1	0	1	NG
14	28	F	TBI	9	1	1	1	NG

Coma recovery scale—revised (CRS-R) total score 23; Hypoxic-Ischemic Brain Injury (HIBI); Ischemic Stroke (I Stroke); Intracerebral haemorrhage (ICH), Subarachnoid haemorrhage (SAH); Traumatic brain injury (TBI); Nasogastric tube (NG); Gastrostomy tube (G).

Conforming to the definition of Nox-T3 swallowing detection, Table 4 shows the number recorded and signal combinations per patient. 82.2% of the detected swallowing acts are made up of the combined three signals, EMG1, EMG2 and apnea, combination A. D is the next most common combination with 6.0%. Then, combinations B and C represent about 4% each. Combination E, G and H represent one percent or less of the combinations. Finally, there was no measurement of the combination F.

**Table 4:** Number of each combination, of TPs, FPs, TNs and FNs per patient as well as sensitivity and specificity per patient.

ID	Swallowing detection					Non-swallowing			Statistical analysis					
	A	B	C	D	E	F	G	H	TP	FP	TN	FN	sens	spec
1	1	2	5	0	0	0	2	0	8	1	349	2	0.8	1
2	21	0	2	0	0	0	0	0	16	3	340	1	0.94	0.99
3	28	0	0	2	0	0	1	0	21	9	329	1	0.95	0.97
4	35	5	2	1	0	0	0	1	29	19	303	9	0.76	0.94
5	1	0	1	0	0	0	0	0	1	2	357	0	1	0.99
6	14	3	0	1	0	0	0	0	15	3	342	0	1	0.99
7	1	0	0	0	0	0	0	0	1	0	359	0	1	1
8	10	1	2	0	0	0	0	1	6	10	338	6	0.5	0.97
9	11	0	0	0	0	0	0	0	8	2	350	0	1	0.99
10	1	1	0	1	0	0	0	0	2	2	355	1	0.67	0.99
11	84	0	0	2	0	0	0	0	72	12	275	1	0.99	0.96
12	3	0	0	2	0	0	0	0	4	1	355	0	1	1
13	11	0	0	0	0	0	0	0	11	0	349	0	1	1
14	11	1	0	8	1	0	0	0	21	0	339	0	1	1

TPs, True positives; FPs, False positives; TNs, True negatives; FNs, False negatives; sens, Sensitivity; spec, Specificity.

According to the proposed protocol and the methodology used to analyze the data, the detection of swallowing by Nox-T3 with sEMG, nasal or tracheal cannulas and RIP belts had an estimated overall sensitivity of 0.95 (95% confidence interval [0.87; 0.99]) and overall specificity of 0.99 (95% confidence interval [0.99; 1]). According to the definition of Nox-T3 swallowing signal, Table 4 presents the number of A-H combinations per patient, the number of TPs, FPs, TNs and FNs for each patient and the within-patient sensitivity and specificity.

**Figure 2:**
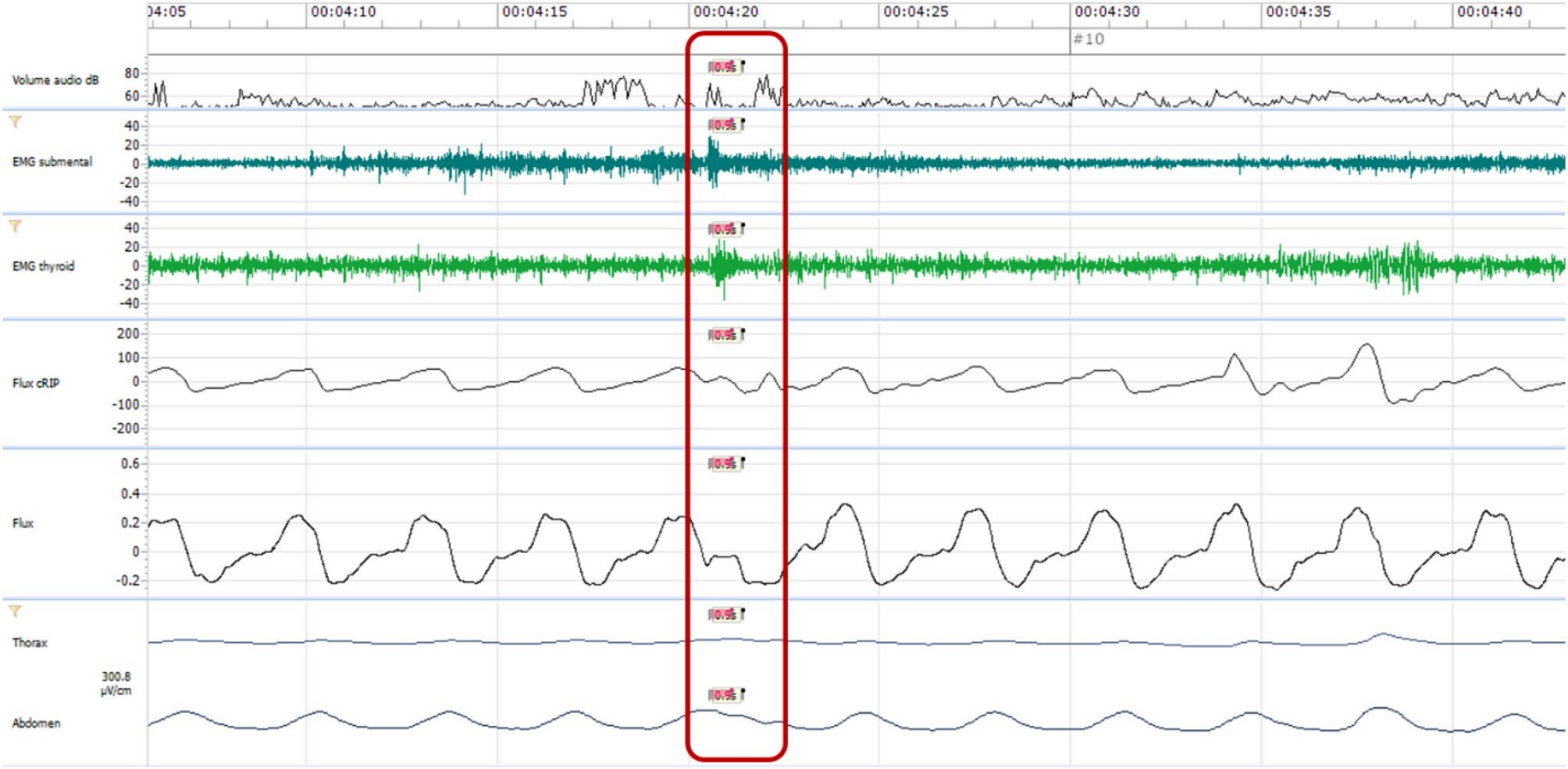
Capture of a swallowing act in patient ID3 at time 1 (00:04:20). Presence of the three signals and clinical detection of the act. The red box localizes the swallowing act.

**Figure 3:**
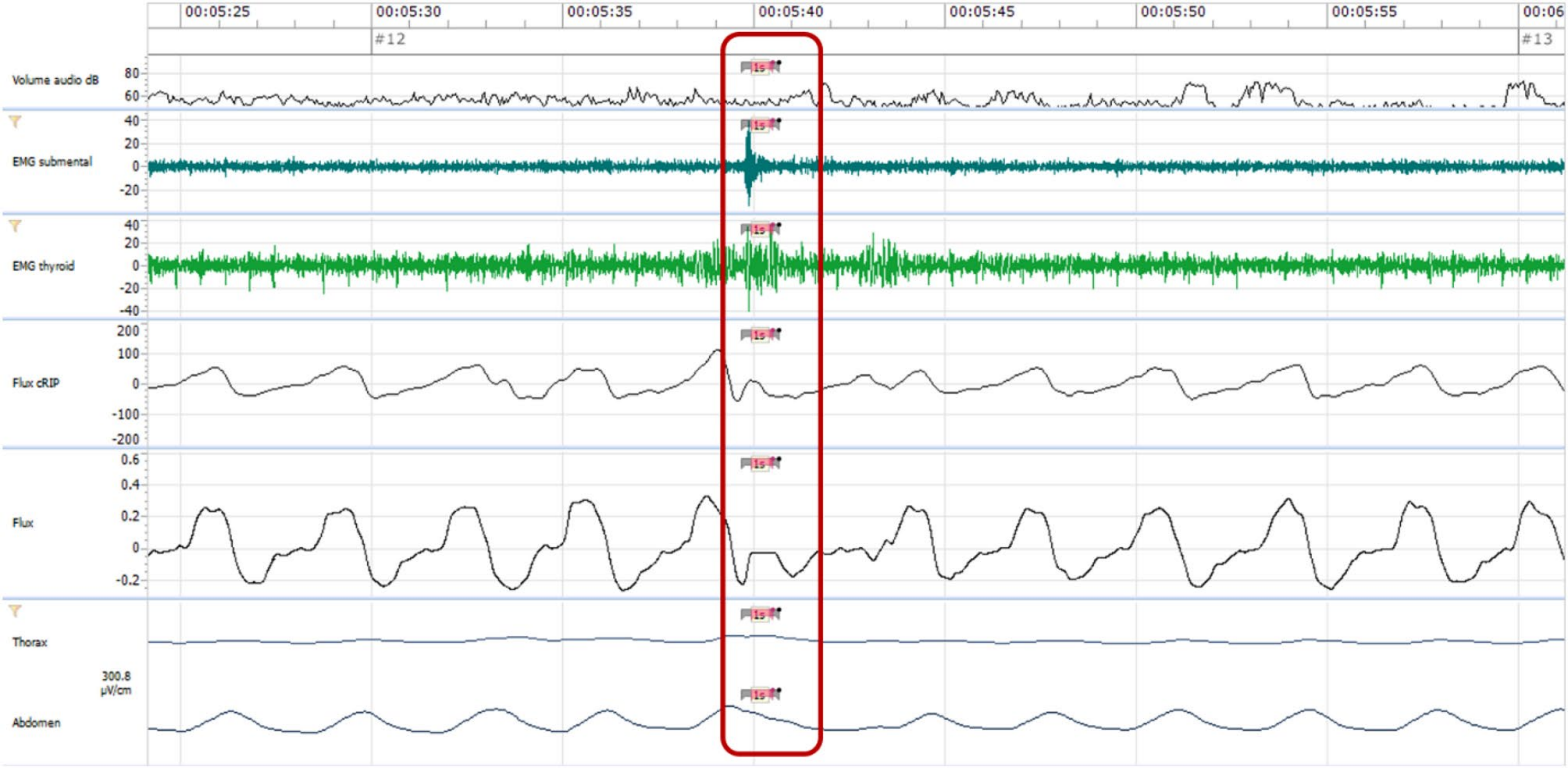
Capture of a swallowing act in patient ID3 at time 2 (00:05:40). Presence of the three signals but no clinical detection. The red box localizes the signaled swallowing act.

The 8 combinations (A-H) were developed for the purpose of the statistical analysis. It is important to consider that it is necessary to analyze the entire tracks of a patient, not only the independent swallowing acts. For example, in Figs. 2 and 3, patient ID3 illustrates two similar swallowing patterns, corresponding to combination A, but opposing clinical results. Indeed, we observed a Nox-T3 signal at time 1 (00:04:20) with the pattern shown in Fig. 2, accompanied by clinical detection of the swallowing act. Then, at time 2 (00:05:40), a very similar signal is seen on the plot (Fig. 3), but this time without clinical swallowing detection. Two similar Nox-T3 signals will then be considered different statistically, i.e. a TP for time 1 and a FP for time 2.

## Discussion

Our results, compared to manual palpation, show that the proposed protocol using Nox-T3 allows non‐invasive detection of swallowing and respiration patterns at the bedside with a sensitivity of 0.95 and a specificity of 0.99, signifying potential clinical use of Nox-T3 for detecting swallowing act in DOC patients.

At present, no diagnostic method has been validated for the objective and normative investigation of swallowing disorders and respiration in DOC patients^20^. The standard objective techniques of VFS and FEES^47,48^, assess swallowing from the presence of aspirations, penetrations, or residues. However, the methods are not fully adapted to DOC patients. VFS needs x-rays, making it unsuitable for repetition and there is a risk of opaque material aspiration^49,50^. Besides, the state of the patient could limit possibility to maintain an adequate position and to cooperate^51^. FEES may be preferential in these patients, since it can be applied at the bedside, repeated and does not imperatively need food or liquid to stimulate swallow^50–52^. However, there is still the issue of patient compliance, as it is necessary that the patient be able to respond to simple orders, which is generally not the case with DOC patients. In addition, neither method evaluates the coordination of breathing with swallowing.

Given the desire for a non-invasive and portable tool, several studies have developed devices capable of measuring swallowing and breathing non-invasively and simultaneously. Different devices, such as the accelerometer^34,43^, force sensing resistor^33^ or microphone^32^, have been used to monitor the movements of the larynx. In our approach, we used sEMG sensors; many studies^41,42^ have shown that sEMG provides reliable information on the timing of muscle contraction and provides dependable screening for dysphagia. It is a non-invasive, low-cost and simple method^53,54^. It does not require the patient's cooperation and is therefore suitable for DOC patients. Concerning respiration coordination, Tarrant and Ellis^39^ showed that the most useful and reliable technique is to record the direct nasal airflow. This method is minimally invasive, allowing access to the mouth during its use, and provides information in real time. However, it is essential to add a second method of respiration recording as some patients are mouth breathers, and there will be no airflow through the nose, although the patient is still breathing. It is therefore necessary to distinguish between mouth breathing and swallowing apnea. Here, for greater accuracy, we decided to supplement the direct airflow recording by recording the respiratory effort with RIP belts, as shown by Moreau-Gaudry and Sabil^55^. Nox-T3 combines the different sensor measures and provides a united tool for swallowing and breathing detection.

Concerning the sensitivity of Nox-T3, the results show that situations where it does not detect a swallowing act (i.e. FNs) are very rare. They are caused by trouble in capturing; as, due to the altered state of consciousness, some patients will be agitated or present autonomic dysfunction^56^, reducing the adhesion of the electrodes and complicating signal capture. These different elements can therefore decrease Nox-T3 sensitivity, but remain a minority. Regarding specificity, the cases of FPs are higher, which tends to decrease specificity. However, the results must be weighed against the fact that clinical palpation is not 100% sensitive. Indeed, it relies on a speech therapist assessing the patient in the acute care setting, with some swallowing acts going undetected even though Nox-T3 clearly detects them, as observed in patient ID3 (see above). In such a case, Nox-T3 does detect an act of swallowing but this creates an erroneous FP as there is no clinical detection. Nox-T3 may then be more suited to the acute care setting without the need of a therapist’s presence during the examination.

As stated above, Nox-T3 seems to have sufficient sensitivity and specificity to detect swallowing and respiration. In addition, there is also qualitative contribution of Nox-T3 analysis. Visualization of the signals brings a lot of information on the swallowing act such as the relationship between muscular and respiratory activity.

**Figure 4:**
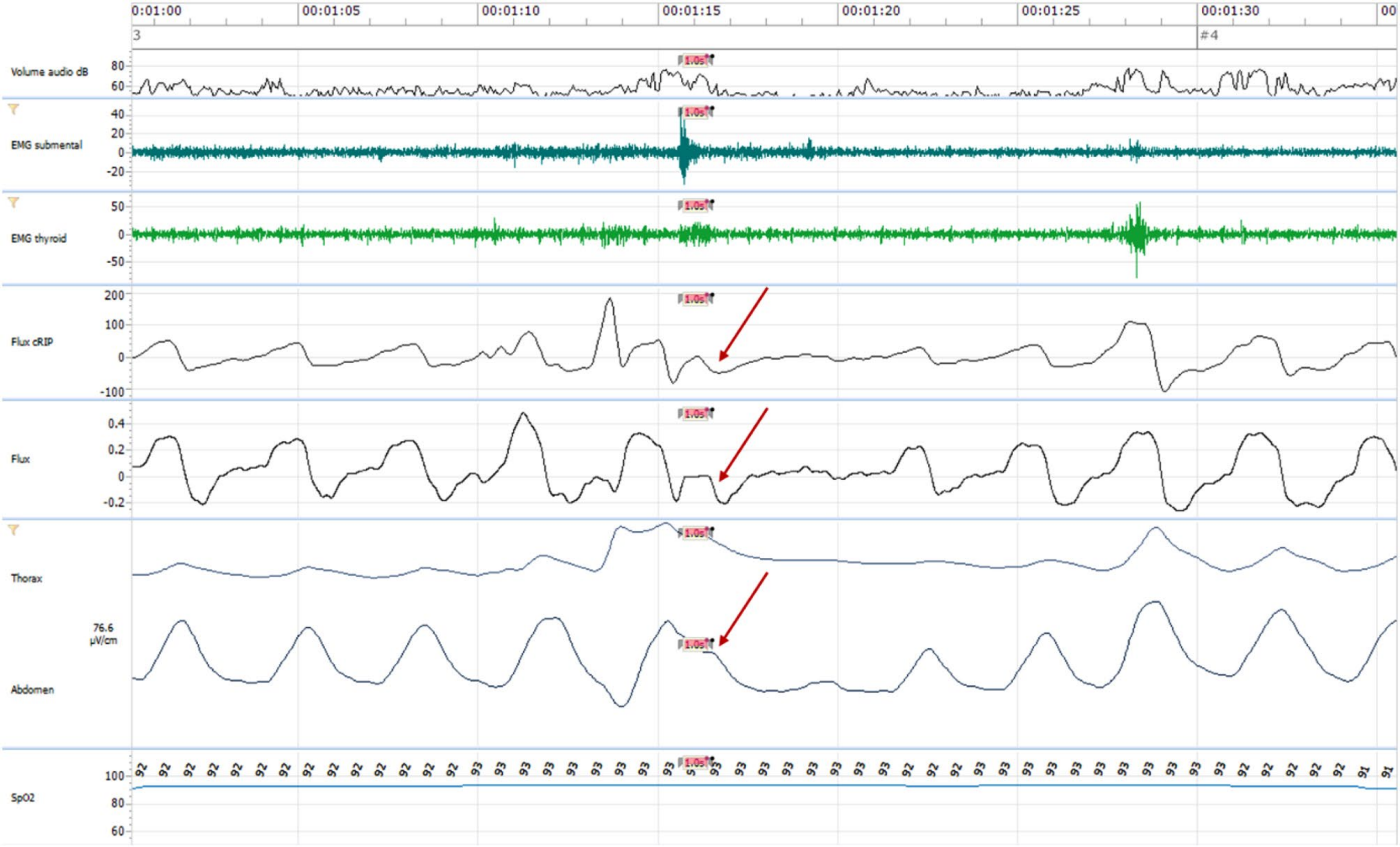
Swallowing apnea followed by expiration. Arrows indicate expiration on the different respiratory tracts.

**Figure 5:**
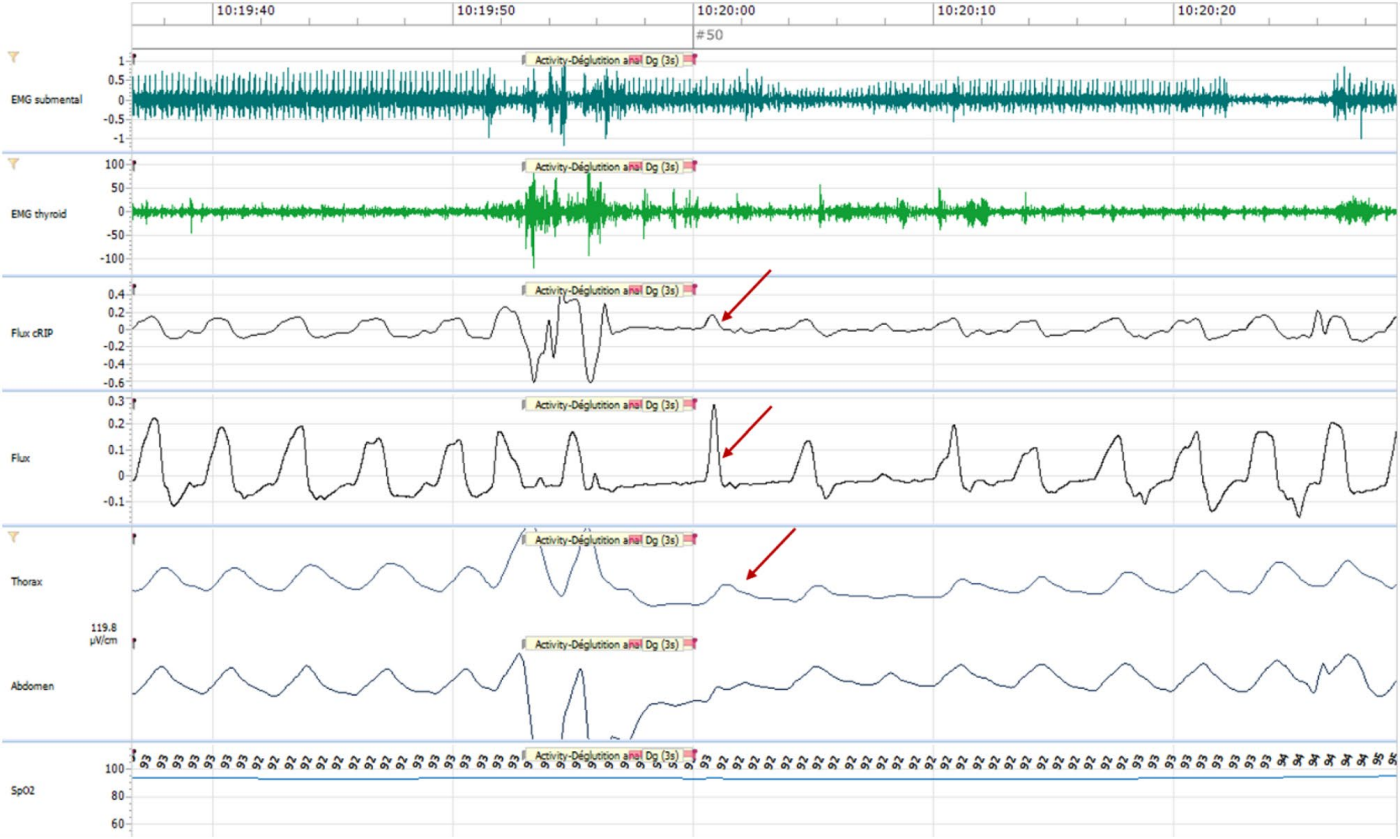
Swallowing apnea followed by inspiration. Arrows indicate inspiration on the different respiratory tracts.

Coordination of breathing contributes to a safer swallow regarding the aspiration-risk. Nox-T3 shows the respiratory frequency and coordination with swallowing, including position of central swallowing apnea in the respiratory cycle. As mentioned before, this apnea is followed physiologically by an expiratory phase^57,58^ (Fig. 4), protecting the airways from aspiration. During dysphagia, an inspiratory phase may follow the apnea (Fig. 5) or the apnea may be completely absent, which tends to increase the risk of aspiration^59^. In addition to the position of the apnea, its duration can also be measured, which may be prolonged during dysphagia^58^.

Regarding muscle signals, a prolonged SM EMG signal before the PL EMG signal may appear. This corresponds to a prolonged oral phase, which increases the bolus preparation time and can be a pathological sign^60^. Submental activity not followed by a laryngeal signal may also be found, twice in patient number one of this study for example. In such cases, there is an oral activity called chewing but no complete swallowing act. This corresponds to reflex chewing without triggering. In the opposite case, where PL EMG activity is observed without oral activity, reflex swallowing takes place (combination B). This occurs without preparation and unconsciously.

The number of swallowing acts in a given time is provided on each trace, allowing an evaluation of swallowing frequency. To be objective, frequency measurement requires recording over a long period of time. Nox-T3 allows this acquisition without the need of a therapist. Frequency can be reduced to up to 1 swallow in 30 min, as in our study, increasing the risk of aspiration^61^. Contrarily, the swallowing frequency can also be increased—either globally over the entire recording or indicated by the presence of multiple swallows. Multiple swallowing is a sign of oral transport disorder^20,60^.

Regarding rehabilitation therapy, in the NRA unit of our hospital, speech therapists use the FOTT technique developed by Kay Coombes^28^ and describe by Hansen and Jakobsen^29^ as a structured concept to assess and treat swallowing and voice disorders. FOTT does not require active participation of the patient and is therefore suitable for DOC patients. At present, there is no objective evaluation for this rehabilitation method and perhaps Nox-T3 could provide this. The graphical representation of the Nox-T3 allows comparing each swallowing act and could highlight pre- and post-rehabilitation differences. By repeating recordings several days apart, Nox-T3 would bring an understanding of the evolution of a patient's swallowing acts, for example by seeing the change in the place of apnea on the breathing cycle curve, modification of coordination between SM and PL muscle or increasing in swallowing frequency. This could also be correlated to the clinical evolution; for example, with a change of food texture or a lung condition or by looking at the amplitude of the EMG signals, it could correlate with clinically observed hypotonia. Importantly, as the device is quite simple to use and does not require much training to use, it would allow continued neurorehabilitation in a tertiary institution.

Finally, in the future, the Nox-T3 could help differentiating between DOC patients. In fact, as stated above, the presence of swallowing act can be an early indication of conscious behaviour. As MCS patients are more likely to recover than patients in UWS; therefore, an accurate diagnosis of consciousness is essential for their optimal medical care. However, differentiating between patients in UWS and MCS still represents a challenge^62^. In this respect, the swallowing abilities of patients with DOC should be systematically assessed and taken into account in DOC diagnosis^23^, therefore the potential usefulness of Nox-T3.

Nonetheless, Nox-T3 use has some limitations for clinical application. As mentioned above, some patients may have autonomic dysfunction that diminishes the adhesion of the electrodes and therefore the capture of the Nox-T3. In addition, reading the plots cannot be done in real time, requiring later analysis. Finally, unlike standard methods, Nox-T3 neither allows direct visualization of the bolus passage nor the aspirations.

Limitations of this study include its monocentric nature with a small number of patients. Indeed, the Acute Neuro-Rehabilitation Unit of the CHUV has only two beds, which limits the study population. It would therefore be interesting to continue exploration of Nox-T3 in larger multi-center cohorts. Future studies are also needed to evaluate the effect of potential predictors such as age, sex or the presence of a tracheotomy on the sensitivity and specificity of Nox-T3 measures. Finally, the use of Nox-T3 could be extended to other types of dysphagic populations.

## Conclusion

Our findings suggest that Nox-T3 could be an adequately sensitive and objective method to detect swallowing and respiration coordination in neurologically impaired patients. In order to validate these results, measurements should be continued and extended to other centers to include more patients. This device can be carried out at the bedside and is non-invasive and relatively quick and easy to perform. The method also allows simultaneous detection of swallowing and breathing and therefore delivers clinically relevant information for rehabilitation. It consequently offers prospects for the quantitative evaluation of rehabilitation, especially by the FOTT.

## Supplementary Information

Supplementary Information.

## Data Availability

All data generated or analysed during this study are included in this published article (and its Supplementary Information files).
