# Supplementary material for: Feasibility study of the Nox-T3 device to detect swallowing and respiration pattern in neurologically impaired patients in the acute phase
Source: Sci Rep. 2023 May 5;13:7325. doi: 10.1038/s41598-023-32628-y (PMC10163003; doi:10.1038/s41598-023-32628-y)
Supplement: Supplementary file 1 — Supplementary Information. [file 41598_2023_32628_MOESM1_ESM.docx]

# **Feasibility study of the Nox-T3 device to detect swallowing and respiration pattern in neurologically impaired patients in the acute phase**

Fanny Theytaz MMed.^1#*^; Aline Vuistiner MSc.^2#^ ; Valérie Schweizer M.D. ^2^ ; Adélie Crépin MSc. ^2^; Kishore Sandu M.D^3^.; Aziz Chaouch BSc.^4^; Lise Piquilloud M.D^5^.; Gianpaolo Lecciso BSc.^6^ , Kay Coombes MSc.^7^ and Karin Diserens M.D^8^.

1. University of Lausanne, CH-1015 Lausanne, Switzerland,
2. Phoniatrics and Speech therapy unit, Lausanne University Hospital (CHUV), Rue du Bugnon, 46, 1011 Lausanne Switzerland
3. Airway Unit, Department of Otorhino-laryngology and Head and Neck Surgery, Lausanne University Hospital (CHUV), Rue du Bugnon, 46, 1011 Lausanne Switzerland
4. Division of Biostatistics, Center for Primary Care and Public Health (Unisanté), University of Lausanne, Lausanne, Switzerland
5. Adult intensive care unit, Lausanne University Hospital (CHUV), Rue du Bugnon, 46, 1011 Lausanne Switzerland
6. Centre d’investigation et de recherche sur le sommeil, Lausanne University Hospital (CHUV), Rue du Bugnon 46, 1011 Lausanne
7. Association for Rehabilitation of Communication and Oral Skills (ARCOS), Malvern Centre, Hatherton Lodge, Avenue Road, Malvern Worcestershire WR14 3AG, United Kingdom
8. Acute neurorehabilitation unit, Neurology service, Department of Clinical Neurosciences, Lausanne University Hospital (CHUV), Rue du Bugnon, 46, 1011 Lausanne, Switzerland

# These authors contributed equally to the work

* Corresponding author, [fanny.theytaz@bluewin.ch](mailto:fanny.theytaz@bluewin.ch)

# **Supplementary Material**

## Detailed procedure for use of Nox-T3 to measure swallowing and respiration activity

**Time 0: Installation of the Nox-T3**

1. Patient preparation: We position the patient according to their possibilities, ideally semi-sitting (45°) in bed or on a chair (90°), maintaining a state of arousal. We record the respiratory flow as per the patient comorbidities.
   1. Patient without tracheotomy: The patient's nose is checked for permeability with a mirror placed under the nasal apertures. If the nose is not permeable, a request for nose care is made to the patient's nurse and if necessary the test is postponed to the following day.
   2. Patient with tracheotomy: if possible, we insert a hermetic cap and we proceed as describe in 1.a. If this is not possible, the tracheotomy cannula is cleaned to be able to perform airflow measurements. If the first two options are not feasible, we attempt to adapt on a case-by-case basis.
2. Nox-T3 Placement (Fig.S1)
   1. The Nox-T3 box is attached at the sternum using tweezers attached to the patient's clothing.
   2. If necessary, the skin is first cleaned with soap and then with disinfectant.
   3. Two pairs of EEG electrodes are filled with conduction gel and attached as in supplementary figure 2:
      1. Submental muscles, bilateral: an electrode on each side on the digastric muscle and/or mylohyoid (black). Do not place the electrodes on the mandibular bone surface.
      2. Peri-laryngeal muscles, unilateral: one electrode on the cricoid arch and one on the ipsilateral thyroid lobe (yellow). Do not place the electrodes at the midline or on the sternocleidomastoid muscle.
   4. The grounding electrode is placed on the clavicle or the iliac crest (green).
3. The nasal cannula is placed at the entrance of the nostrils. For tracheotomy patients, if the withdrawal process is sufficiently advanced for the insertion of a hermetic plug, a nasal recording is performed. If the tracheostomy cannot be closed, an adapter on the tracheostomy tube is used.
4. The saturometer is installed on one of the patient's indices.
5. We observe the patient's breathing and install one of the RIP belts over the patient's clothing at a level of maximum amplitude; corresponding most of the time either to the nipples or umbilicus.
6. Switch on the Nox-T3 recording and do not interrupt over times 1, 2 and 3.


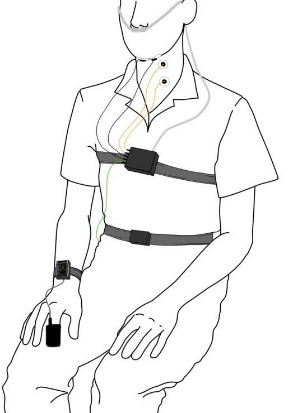
 **Fig. S1**: **Nox-T3 placement**. SM electrodes are black, PL electrodes yellow and the grounding electrode green.


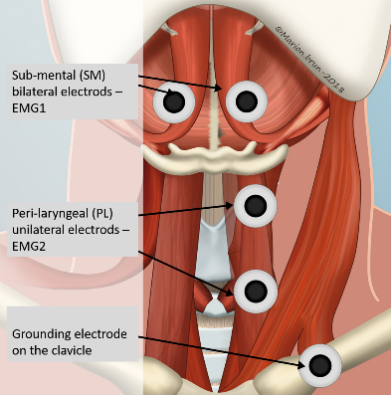
 **Fig. S2**: **Electrode positioning**

**Time 1: Recording and clinical observation – 10 minutes**

At the start of Time 1, the patient's respiratory rate and saturation are recorded.

- We attempt to maintain arousal without stimulating swallowing.
- Laryngeal palpation will help detect the number of attempts of the swallowing reflex.
- When swallowing occurs, the speech therapist mentions it aloud, recording it on the Nox-Microphone, which is repeated throughout time steps 1-3.

**Time 2: Stimulation and recording**

- A rehabilitation session according to the FOTT (22,26), with an adapted therapeutic food trial, if deemed clinically possible by the speech therapist (iced sorbet, crushed ice, syrup or liquid honey consistency, yogurt, banana or bread).
- We then perform Global clinical assessment of swallowing and Functional Oral Intake Scale (FOIS) rating (25).

**Time 3: Recording and clinical observation at 10 minutes**

At the beginning of Time 3, we note the patient's respiratory rate and saturation.

- We attempt to maintain arousal without stimulation of swallowing and if possible with the same settings as time 1 (patient position, external environment, etc.).
- Laryngeal palpation to detect the number of attempts of the swallowing reflex. When swallowing occurs, mention it aloud.

**Time 4: Storage and Nox-T3 sanitizing**

- The single-use material is disposed of
- The Nox-T3, EMG wires, pulse oximeter and cables are disinfected using SteriWipes-C wipes.
- The differential pressure sensor is disinfected as recommended by the manufacturer using Mikrozid (25 g ethanol (94 %), 35 g 1-Propanol).

**Time 5: Nox-T3 signal reading**

We transfer data from Nox-T3 to the Noxturnal software. All detectable swallowing acts and time frames are recorded and subjected to numerical analysis.

**Table S1 : Extraction of Nox-T3 tracts data:** for each patient during the 30 minutes of recording, the times at which an EMG1, EMG2 or apnea signal is recorded . Clinical signal time, correspond to the timing of palpation of a swallowing act by the speech therapist.

| Patient ID | Clinical signal time | Start of EMG 1 signal | Start of EMG 2 signal | Apnea time |
| --- | --- | --- | --- | --- |
| ID1 | 0:1:30,5 | 0:1:27,1 | - | 00:01:31 |
|  | 0:3:29,5 | 0:3:27,8 | - | - |
|  | 0:9:39,9 | 0:9:37,9 | - | - |
|  | 0:13:44,3 | 0:13:44,2 | - | 00:13:43 |
|  | 0:14:48,4 | 0:14:47,2 | - | 00:14:47 |
|  | 0:16:37,8 | - | 0:16:36,6 | 00:16:36 |
|  | 0:17:55,4 | 0:17:53,6 | - | 00:17:53 |
|  | 0:18:18,8 | 0:18:16,9 | - | 00:18:17 |
|  | 0:20:16,2 | 0:20:15,1 | 0:19:55,6 | 00:20:15 |
|  | 0:22:7,5 | 0:22:6,4 | - | 00:22:06 |
| ID2 | 0:2:39,9 | 0:2:37,3 | 0:2:37,3 | 00:02:39 |
|  | 0:7:35,2 | 0:7:32,9 | 0:7:33,8 | 00:07:34 |
|  | 0:14:59,2 | 0:14:53,5 | - | 00:14:55 |
|  | 0:15:32,4 | 0:15:29,7 | 0:15:30,9 | 00:15:31 |
|  | 0:16:32,2 | 0:16:29,2 | 0:16:27,9 | 00:16:31 |
|  | - | 0:17:26,1 | - | 00:17:27 |
|  | 0:17:45,0 | 0:17:43,1 | 0:17:43,7 | 00:17:43 |
|  | 0:19:33,8 | 0:19:31,3 | 0:19:31,5 | 00:19:31 |
|  | 0:21:35,6 | 0:21:32,7 | 0:21:33,5 | 00:21:33 |
|  | 0:22:51,7 | 0:22:48,9 | 0:22:50,5 | 00:22:50 |
|  | 0:24:14,1 | 0:24:12,1 | 0:24:12,1 | 00:24:12 |
|  | 0:24:46,7 | 0:24:45,3 | 0:24:45,3 | 00:24:45 |
|  | 0:25:16,2 | 0:25:13,9 | 0:25:13,9 | 00:25:14 |
|  | 0:25:29,7 | 0:25:26,6 | 0:25:26,6 | 00:25:28 |
|  | 0:26:21,8 | 0:26:18,2 | 0:26:19,5 | 00:26:20 |
|  | - | 0:26:36,7 | 0:26:36,7 | 00:26:37 |
|  | 0:26:52,9 | 0:26:50,3 | 0:26:49,8 | 00:26:51 |
|  | 0:27:5,8 | 0:27:2,7 | 0:27:2,7 | 00:27:04 |
|  | 0:28:28,7 | 0:28:25,5 | 0:28:25,5 | 00:28:27 |
|  | 0:31:7,8 | 0:31:4,8 | 0:31:4,8 | 00:31:06 |
|  | 0:31:52,2 | 0:31:49,5 | 0:31:50,9 | 00:31:51 |
|  | 0:32:23,4 | 0:32:21,4 | 0:32:21,2 | 00:32:22 |
|  | 0:33:5,6 | 0:33:3,4 | 0:33:4,3 | 00:33:04 |
| ID3 | - | 0:1:8,5 | 0:1:8,5 | 00:01:08 |
|  | 0:2:0,9 | 0:1:59,8 | 0:1:59,8 | 00:01:59 |
|  | 0:4:15,2 | 0:4:13,4 | 0:4:13,4 | 00:04:13 |
|  | - | 0:5:32,6 | 0:5:32,7 | 00:05:32 |
|  | - | 0:6:53,8 | 0:6:53,2 | 00:06:54 |
|  | 0:8:35,2 | 0:8:31,8 | 0:8:30,8 | 00:08:33 |
|  | 0:12:6,8 | 0:12:5,8 | 0:12:5,8 | 00:12:06 |
|  | 0:12:51,2 | 0:12:49,8 | 0:12:49,8 | - |
|  | 0:13:2,8 | 0:13:1,5 | 0:13:1,7 | 00:13:01 |
|  | 0:13:42,9 | 0:13:42,3 | 0:13:42,3 | 00:13:42 |
|  | 0:14:17,5 | 0:14:16,4 | 0:14:16,4 | 00:14:16 |
|  | - | 0:14:20,9 | 0:14:21,5 | 00:14:21 |
|  | - | 0:15:1,1 | 0:15:1,2 | 00:15:01 |
|  | 0:15:22,2 | 0:15:21,1 | 0:15:21,1 | 00:15:21 |
|  | - | 0:15:33,9 | 0:15:33,9 | 00:15:34 |
|  | 0:15:58,1 | 0:15:56,7 | 0:15:56,8 | 00:15:56 |
|  | 0:16:51,8 | 0:16:50,3 | 0:16:50,5 | 00:16:50 |
|  | 0:17:44,8 | 0:17:43,5 | 0:17:43,5 | 00:17:43 |
|  | - | 0:18:2,6 | 0:18:2,5 | 00:18:02 |
|  | 0:18:9,5 | 0:18:8,2 | 0:18:8,2 | 00:18:08 |
|  | 0:18:40,2 | 0:18:38,6 | 0:18:38,6 | 00:18:38 |
|  | 0:19:9,3 | 0:19:7,8 | 0:19:7,4 | - |
|  | 0:19:24,2 | 0:19:22,9 | 0:19:22,8 | 00:19:23 |
|  | - | 0:19:39,5 | 0:19:39,5 | 00:19:39 |
|  | - | 0:19:59,8 | 0:19:59,7 | 00:20:00 |
|  | 0:20:33,8 | 0:20:32,4 | 0:20:32,4 | 00:30:32 |
|  | 0:22:6,2 | 0:22:3,8 | - | - |
|  | 0:23:37,6 | 0:23:36,1 | 0:23:36,2 | 00:23:36 |
|  | 0:24:13,8 | 0:24:12,2 | 0:24:12,2 | 00:24:12 |
|  | 0:27:6,9 | 0:27:5,5 | 0:27:5,5 | 00:27:05 |
|  | 0:29:30,1 | 0:29:28,7 | 0:29:28,1 | 00:29:28 |
| ID4 | 0:2:52,9 | 0:2:50,5 | 0:2:50,4 | 00:02:50 |
|  | 0:3:22,0 | 0:3:17,8 | 0:3:17,5 | 00:03:19 |
|  | 0:4:5,2 | 0:3:55,2 | 0:3:55,1 | 00:04:00 |
|  | 0:5:9,0 | 0:5:4,5 | 0:5:5,1 | 00:05:05 |
|  | 0:9:6,6 | 0:8:55,2 | 0:8:57,1 | 00:09:04 |
|  | 0:9:13,9 | 0:9:9,1 | 0:9:9,1 | - |
|  | 0:10:55,4 | 0:10:40,1 | 0:10:39,4 | 00:10:52 |
|  | 0:11:3,9 | 0:10:57,1 | 0:10:57,3 | 00:10:58 |
|  | 0:11:52,1 | 0:11:50,8 | 0:11:51,4 | 00:11:50 |
|  | - | 0:12:23,8 | 0:12:24,8 | 00:12:24 |
|  | 0:12:36,8 | 0:12:34,6 | 0:12:35,2 | 00:12:34 |
|  | 0:13:46,6 | - | 0:13:44,1 | 00:13:45 |
|  | 0:14:17,4 | 0:14:11,8 | 0:14:13,5 | 00:14:14 |
|  | 0:15:0,9 | 0:14:49,1 | 0:14:52,9 | 00:14:56 |
|  | 0:15:41,1 | 0:15:32,2 | 0:15:33,5 | 00:15:37 |
|  | 0:15:50,5 | 0:15:41,7 | 0:15:41,7 | 00:15:49 |
|  | 0:16:45,4 | 0:16:36,4 | - | - |
|  | 0:17:18,1 | 0:17:10,2 | 0:17:13,3 | 00:17:15 |
|  | 0:17:43,4 | 0:17:40,2 | 0:17:42,7 | 00:17:42 |
|  | 0:18:13,4 | 0:18:7,6 | 0:18:8,7 | 00:18:11 |
|  | 0:19:14,2 | 0:19:10,4 | 0:19:11,3 | 00:19:12 |
|  | 0:19:45,6 | 0:19:43,8 | - | 00:19:44 |
|  | 0:20:34,2 | - | 0:20:29,9 | 00:20:31 |
|  | 0:20:43,6 | 0:20:39,8 | 0:20:41,4 | 00:20:42 |
|  | 0:20:50,9 | 0:20:46,2 | 0:20:46,5 | 00:20:48 |
|  | 0:22:0,4 | - | 0:21:53,8 | 00:21:58 |
|  | 0:23:28,4 | 0:23:21,9 | 0:23:22,1 | 00:23:25 |
|  | 0:23:31,2 | - | - | - |
|  | 0:24:1,3 | 0:23:56,2 | 0:23:58,7 | 00:23:59 |
|  | 0:24:24,0 | 0:24:18,5 | 0:24:18,1 | 00:24:22 |
|  | 0:25:8,9 | 0:25:1,3 | 0:25:4,3 | 00:25:06 |
|  | 0:25:20,8 | 0:25:15,4 | 0:25:16,3 | 00:25:18 |
|  | 0:26:18,9 | 0:26:10,5 | 0:26:15,6 | 00:26:16 |
|  | 0:26:31,2 | 0:26:27,5 | 0:26:27,5 | 00:26:28 |
|  | 0:26:48,2 | - | 0:26:57,4 | 00:26:55 |
|  | 0:27:6,3 | 0:27:2,5 | 0:27:3,1 | 00:27:04 |
|  | - | 0:27:10,6 | 0:27:12,9 | 00:27:12 |
|  | 0:27:55,4 | 0:27:53,4 | 0:27:48,3 | 00:27:53 |
|  | 0:27:59,8 | 0:27:57,2 | 0:27:58,8 | 00:27:53 |
|  | 0:28:51,6 | - | 0:28:50,3 | 00:28:50 |
|  | 0:28:53,5 | 0:28:53,2 | 0:28:53,1 | 00:28:50 |
|  | 0:28:56,1 | 0:28:55,7 | 0:28:55,6 | 00:28:50 |
|  | 0:29:26,3 | 0:29:21,9 | 0:29:25,7 | 00:29:25 |
|  | 0:29:28,2 | 0:29:21,9 | 0:29:27,6 | 00:29:25 |
|  | 0:29:30,6 | 0:29:21,9 | - | 00:29:25 |
| ID5 | 0:29:32,9 | 0:29:21,9 | 0:29:32,2 | 00:29:25 |
|  | - | 0:13:18,6 | - | 00:13:19 |
| ID6 | 0:29:54,3 | 0:29:52,2 | 0:29:52,3 | 00:29:52 |
|  | 0:7:12,1 | 0:7:10,4 | 0:7:10,9 | - |
|  | - | 0:9:33,6 | 0:9:34,1 | 00:09:34 |
|  | 0:11:51,9 | 0:11:50,1 | 0:11:50,6 | 00:11:50 |
|  | 0:13:21,4 | 0:12:19,6 | 0:13:20,3 | 00:13:20 |
|  | 0:14:38,2 | 0:14:36,5 | 0:14:37,0 | 00:14:36 |
|  | 0:15:51,8 | 0:15:50,2 | 0:15:50,6 | 00:15:50 |
|  | 0:16:34,4 | 0:16:32,5 | 0:16:32,7 | 00:16:32 |
|  | - | 0:16:40,4 | 0:16:40,7 | 00:16:40 |
|  | 0:17:3,2 | - | 0:17:1,7 | 00:17:01 |
|  | 0:17:12,8 | 0:17:10,6 | 0:17:11,2 | 00:17:11 |
|  | - | 0:17:19,9 | 0:17:20,6 | 00:17:20 |
|  | 0:18:17,9 | - | 0:18:16,4 | 00:18:16 |
|  | 0:19:41,7 | 0:19:40,2 | 0:19:40,5 | 00:19:40 |
|  | 0:21:3,9 | 0:21:1,6 | 0:21:1,9 | 00:21:02 |
|  | 0:23:50,4 | - | 0:23:48,8 | 00:23:49 |
|  | 0:27:8,1 | 0:27:6,4 | 0:27:6,7 | 00:27:06 |
|  | 0:29:20,8 | 0:29:18,9 | 0:29:19,3 | 00:29:19 |
| ID7 | 0:29:45,8 | 0:29:44,3 | 0:29:44,3 | 00:29:45 |
| ID8 | 0:1:43,2 | 0:1:33,5 | 0:1:34,2 | - |
|  | 0:1:55,4 | 0:1:47,7 | 0:1:47,9 | 00:01:47 |
|  | - | 0:5:25,2 | 0:5:27,4 | 00:05:25 |
|  | 0:7:21,9 | - | - | - |
|  | - | 0:9:52,2 | 0:9:53,7 | 00:09:47 |
|  | 0:11:43,8 | 0:11:35,1 | 0:11:34,7 | 00:11:40 |
|  | 0:12:47,9 | 0:12:40,5 | 0:12:41,1 | 00:12:43 |
|  | 0:13:27,8 | 0:13:24,2 | - | 00:13:24 |
|  | 0:14:53,2 | 0:14:51,8 | - | 00:14:52 |
|  | 0:15:32,5 | 0:15:29,8 | 0:15:30,1 | 00:15:31 |
|  | 0:15:58,2 | 0:15:54,1 | 0:15:54,8 | 00:15:55 |
|  | 0:16:29,3 | 0:16:23,2 | 0:16:24,4 | 00:16:24 |
|  | 0:17:11,3 | 0:17:5,1 | 0:17:6,6 | 00:17:08 |
|  | 0:25:24,8 | 0:25:16,1 | 0:25:18,3 | 00:25:18 |
| ID9 | 0:0:16,3 | 0:0:14,5 | 0:0:14,4 | 00:00:14 |
|  | 0:5:18,1 | 0:5:16,2 | 0:5:16,2 | 00:05:16 |
|  | 0:5:42,5 | 0:5:41,1 | 0:5:41,3 | 00:05:41 |
|  | 0:9:49,3 | 0:9:47,8 | 0:9:47,7 | 00:09:47 |
|  | - | 0:12:9,1 | 0:12:9,3 | 00:12:09 |
|  | - | 0:13:58,2 | 0:13:58,2 | 00:13:58 |
|  | 0:16:56,5 | 0:16:55,5 | 0:16:54,4 | 00:16:54 |
|  | 0:19:51,4 | 0:19:49,5 | 0:19:49,5 | 00:19:49 |
|  | 0:22:51,3 | 0:22:50,2 | 0:22:50,2 | 00:22:50 |
|  | 0:27:17,8 | 0:27:16,4 | 0:27:16,2 | 00:27:16 |
|  | 0:30:4,5 | 0:30:1,4 | 0:30:2,2 | 00:30:03 |
| ID10 | 0:8:31,6 | - | 0:8:28,2 | 00:08:29 |
|  | 0:11:33,4 | 0:11:26,9 | 0:11:27,1 | - |
|  | 0:19:43,2 | 0:19:39,3 | 0:19:33,4 | 00:19:41 |
| ID11 | 0:1:40,6 | 0:1:37,9 | 0:1:38,6 | 00:01:39 |
|  | 0:1:48,2 | 0:1:45,3 | 0:1:45,4 | 00:01:46 |
|  | - | 0:1:51,4 | 0:1:52,2 | 00:01:52 |
|  | 0:2:8,2 | 0:2:7,4 | 0:2:7,2 | 00:02:07 |
|  | 0:2:45,5 | 0:2:44,7 | 0:2:44,7 | 00:02:44 |
|  | 0:5:10,6 | 0:5:8,8 | 0:5:8,7 | 00:05:08 |
|  | 0:5:13,7 | 0:5:11,3 | 0:5:11,4 | 00:05:11 |
|  | 0:5:28,6 | 0:5:21,3 | 0:5:21,6 | 00:05:26 |
|  | 0:5:44,1 | 0:5:43,3 | 0:5:43,8 | 00:05:43 |
|  | 0:6:36,9 | 0:6:34,1 | 0:6:34,2 | 00:06:35 |
|  | 0:6:54,5 | 0:6:48,8 | 0:6:50,2 | - |
|  | 0:7:11,2 | 0:7:9,6 | 0:7:9,5 | 00:07:09 |
|  | 0:7:31,9 | 0:7:29,5 | 0:7:29,7 | 00:07:30 |
|  | 0:8:5,3 | 0:8:3,9 | 0:8:4,7 | 00:08:04 |
|  | 0:8:32,7 | 0:8:31,1 | 0:8:31,0 | 00:08:31 |
|  | 0:9:18,3 | 0:9:14,9 | 0:9:14,9 | 00:09:15 |
|  | 0:10:12,7 | 0:10:11,8 | 0:10:11,7 | 00:10:12 |
|  | 0:10:25,5 | 0:10:23,9 | 0:10:24,1 | 00:10:24 |
|  | 0:10:38,9 | 0:10:37,8 | 0:10:37,7 | 00:10:37 |
|  | 0:11:9,4 | 0:11:5,6 | 0:11:5,5 | - |
|  | 0:11:19,3 | 0:11:16,1 | 0:11:16,4 | 00:11:17 |
|  | 0:11:45,1 | 0:11:42,5 | 0:11:42,5 | 00:11:44 |
|  | 0:12:2,1 | 0:11:59,5 | 0:11:59,7 | 00:12:00 |
|  | 0:12:9,2 | 0:12:7,7 | 0:12:7,7 | 00:12:08 |
|  | 0:12:22,3 | 0:12:21,4 | 0:12:21,8 | 00:12:21 |
|  | 0:12:47,1 | 0:12:45,7 | 0:12:45,9 | 00:12:46 |
|  | - | 0:13:5,9 | 0:13:7,3 | 00:13:07 |
|  | - | 0:13:14,6 | 0:13:15,2 | 00:13:16 |
|  | 0:13:25,5 | 0:13:24,4 | 0:13:24,4 | 00:13:24 |
|  | 0:13:44,3 | 0:13:43,2 | 0:13:43,3 | 00:13:43 |
|  | 0:13:52,3 | 0:13:51,4 | 0:13:51,3 | 00:13:51 |
|  | 0:14:4,9 | 0:14:3,5 | 0:14:3,4 | 00:14:03 |
|  | 0:14:7,3 | 0:14:5,6 | 0:14:5,8 | 00:14:06 |
|  | - | 0:14:10,5 | 0:14:10,5 | 00:14:10 |
|  | 0:14:14,5 | 0:14:12,8 | 0:14:12,9 | 00:14:13 |
|  | 0:14:22,9 | 0:14:21,2 | 0:14:21,6 | 00:14:22 |
|  | - | 0:14:27,1 | 0:14:27,1 | 00:04:27 |
|  | 0:14:32,7 | 0:14:32,6 | 0:14:32,6 | 00:14:32 |
|  | 0:14:37,7 | 0:14:35,6 | 0:14:35,7 | 00:14:35 |
|  | 0:14:48,6 | 0:14:47,1 | 0:14:47,3 | 00:14:47 |
|  | 0:14:55,1 | 0:14:53,2 | 0:14:53,4 | 00:14:53 |
|  | - | 0:15:4,9 | 0:15:5,2 | 00:15:05 |
|  | 0:15:15,1 | 0:15:14,7 | 0:15:14,7 | 00:15:14 |
|  | - | 0:15:23,3 | 0:15:23,5 | 00:15:23 |
|  | 0:15:37,5 | 0:15:36,2 | 0:15:36,3 | 00:15:36 |
|  | 0:15:49,5 | 0:15:48,1 | 0:15:48,2 | 00:15:48 |
|  | 0:16:14,6 | 0:16:11,7 | 0:16:11,3 | 00:16:13 |
|  | 0:16:36,5 | 0:16:34,4 | 0:16:35,1 | 00:16:35 |
|  | 0:16:54,5 | 0:16:52,7 | 0:16:53,8 | 00:16:53 |
|  | 0:17:18,5 | 0:17:17,6 | 0:17:16,1 | 00:17:17 |
|  | 0:17:47,7 | 0:17:45,8 | 0:17:46,3 | 00:17:46 |
|  | - | 0:17:51,5 | 0:17:51,6 | 00:17:51 |
|  | 0:18:6,3 | 0:18:5,7 | 0:18:5,6 | 00:18:05 |
|  | 0:18:20,7 | 0:18:19,7 | 0:18:19,5 | 00:18:19 |
|  | - | 0:18:31,2 | 0:18:31,1 | 00:18:31 |
|  | - | 0:18:37,7 | 0:18:37,7 | 00:18:37 |
|  | 0:18:55,7 | 10:18:54,3 | 0:18:54,2 | 00:18:54 |
|  | 0:19:10,2 | 0:19:8,4 | 0:19:8,4 | 00:19:08 |
|  | 0:19:21,5 | 0:19:20,2 | 0:19:20,5 | 00:19:20 |
|  | 0:19:31,7 | 0:19:30,6 | 0:19:30,6 | 00:19:30 |
|  | 0:19:56,5 | 0:19:55,2 | 0:19:54,9 | 00:19:55 |
|  | 0:20:24,7 | 0:20:23,3 | 0:20:22,8 | 00:20:23 |
|  | 0:21:4,7 | 0:21:2,8 | 0:21:3,8 | 00:21:03 |
|  | 0:21:41,1 | 0:21:38,3 | 0:21:38,4 | 00:21:38 |
|  | 0:22:1,9 | 0:22:1,3 | 0:22:0,9 | 00:22:01 |
|  | 0:22:9,4 | 0:22:8,2 | 0:22:8,4 | 00:22:08 |
|  | 0:22:20,6 | 0:22:19,4 | 0:22:19,3 | 00:22:19 |
|  | 0:22:26,7 | 0:22:25,1 | 0:22:24,9 | 00:22:25 |
|  | 0:22:41,6 | 0:22:38,9 | 0:22:38,7 | 00:22:39 |
|  | 0:22:51,8 | 0:22:48,5 | 0:22:48,4 | 00:22:49 |
|  | 0:22:57,6 | 0:22:56,6 | 0:22:56,3 | 00:22:56 |
|  | 0:23:4,4 | 0:23:3,2 | 0:23:2,8 | 00:23:03 |
|  | 0:23:23,3 | 0:23:21,9 | 0:23:21,7 | 00:23:22 |
|  | 0:23:49,9 | 0:23:48,8 | 0:23:48,5 | 00:23:49 |
|  | 0:23:54,5 | 0:23:53,7 | 0:23:53,8 | 00:23:53 |
|  | 0:24:18,5 | 0:24:17,1 | 0:24:17,1 | 00:24:17 |
|  | 0:24:50,1 | 0:24:48,5 | 0:24:48,1 | 00:24:48 |
|  | 0:25:5,2 | 0:25:3,7 | 0:25:3,7 | 00:25:04 |
|  | 0:25:29,4 | 0:25:28,5 | 0:25:28,0 | 00:25:28 |
|  | 0:26:15,1 | 0:26:13,6 | 0:26:13,5 | 00:26:14 |
|  | 0:26:41,1 | 0:26:40,4 | 0:26:40,5 | 00:26:40 |
|  | 0:27:15,1 | 0:27:13,9 | 0:27:13,8 | 00:27:14 |
|  | 0:27:45,9 | 0:27:45,3 | 0:27:45,1 | 00:27:45 |
|  | 0:28:10,8 | 0:28:9,7 | 0:28:9,7 | 00:28:10 |
|  | 0:28:17,4 | 0:28:15,8 | 0:28:16,2 | 00:28:16 |
|  | - | 0:28:48,5 | 0:28:48,5 | 00:28:49 |
| ID12 | 0:10:5,1 | 0:10:3,5 | 0:10:3,5 | - |
|  | 0:11:56,7 | 0:11:55,4 | 0:11:55,6 | - |
|  | 0:13:37,8 | 0:13:34,7 | 0:13:35,3 | 00:13:35 |
|  | 0:15:47,4 | 0:15:46,3 | 0:15:46,2 | 00:15:46 |
|  | - | 0:18:33,5 | 0:18:33,6 | 00:18:34 |
| ID13 | 0:0:37,7 | 0:0:35,9 | 0:0:36,2 | 00:00:36 |
|  | 0:4:40,8 | 0:4:39,3 | 0:4:39,6 | 00:04:40 |
|  | 0:8:34,8 | 0:8:33,6 | 0:8:33,6 | 00:08:33 |
|  | 0:9:11,7 | 0:9:10,6 | 0:9:10,8 | 00:09:10 |
|  | 0:11:52,4 | 0:11:51,1 | 0:11:51,1 | 00:11:51 |
|  | 0:13:8,7 | 0:13:7,3 | 0:13:7,7 | 00:13:08 |
|  | 0:14:46,5 | 0:14:44,7 | 0:14:44,6 | 00:14:44 |
|  | 0:17:2,2 | 0:17:1,1 | 0:17:1,2 | 00:17:01 |
|  | 0:18:21,8 | 0:18:20,3 | 0:18:20,5 | 00:18:20 |
|  | 0:23:44,4 | 0:23:41,3 | 0:23:41,4 | 00:23:41 |
|  | 0:26:36,1 | 0:26:34,5 | 0:26:34,5 | 00:26:34 |
| ID14 | 0:2:5,2 | 0:2:3,4 | 0:2:3,1 | - |
|  | 0:3:52,7 | 0:3:50,5 | 0:3:50,1 | 00:03:51 |
|  | 0:4:50,8 | 0:4:47,8 | 0:4:48,2 | 00:04:48 |
|  | 0:5:57,2 | 0:5:56,1 | 0:5:55,7 | - |
|  | 0:7:9,9 | 0:7:7,7 | 0:7:7,5 | 00:07:08 |
|  | 0:8:36,4 | - | 0:8:34,6 | - |
|  | 0:10:38,4 | - | 0:10:36,4 | 00:10:37 |
|  | 0:11:22,3 | 0:11:21,6 | 0:11:21,6 | 00:11:21 |
|  | 0:12:3,4 | 0:12:1,5 | 0:12:1,5 | 00:12:01 |
|  | 0:12:34,7 | 0:12:32,9 | 0:12:33,1 | 00:12:33 |
|  | 0:13:44,2 | 0:13:43,4 | 0:13:43,4 | - |
|  | 0:14:20,2 | 0:14:18,8 | 0:14:18,8 | 00:14:19 |
|  | 0:15:26,2 | 0:15:24,6 | 0:15:24,6 | 00:15:25 |
|  | 0:16:36,7 | 0:16:34,1 | 0:16:34,8 | 00:16:35 |
|  | 0:17:31,3 | 0:17:29,7 | 0:17:29,5 | 00:17:30 |
|  | 0:18:43,1 | 0:18:42,0 | 0:18:42,0 | - |
|  | 0:20:4,4 | 0:20:3,1 | 0:20:3,2 | 00:20:03 |
|  | 0:20:41,9 | 0:20:40,8 | 0:20:40,6 | - |
|  | 0:21:43,4 | 0:21:42,2 | 0:21:42,4 | - |
|  | 0:22:21,8 | 0:22:19,8 | 0:22:19,8 | - |
|  | 0:24:48,5 | 0:24:46,6 | 0:24:46,6 | - |
